# Disturbance Observation and Suppression in an Airborne Electro-Optical Stabilized Platform Based on a Generalized High-Order Extended State Observer

**DOI:** 10.3390/s24113629

**Published:** 2024-06-04

**Authors:** Lu Wang, Xiantao Li, Zhanmin Zhou, Yuzhang Liu, Zongyuan Yang, Shitao Zhang, Chong Li

**Affiliations:** 1 Key Laboratory of Airborne Optical Imaging and Measurement, Changchun Institute of Optics, Fine Mechanics and Physics, Chinese Academy of Sciences, Changchun 130033, China; 2 University of Chinese Academy of Sciences, No. 19, Yuquan Rd, Beijing 100049, China; 3 Wuhan Second Ship Design and Research Institute, Wuhan 430060, China; 4 Equipment Department of China PLA Air Force in Changchun Area Military Representative Office, Changchun 130062, China

**Keywords:** generalized high-order extended state observer, airborne electro-optical stabilized platform, sinusoidal periodic disturbance

## Abstract

Active disturbance rejection control (ADRC) is widely used in airborne optoelectronic stabilization platforms due to its minimal reliance on the mathematical model of the controlled object. The extended state observer (ESO) is the core of ADRC, which treats internal parameter variations and external disturbances as total disturbances, observes the disturbances as extended states, and then compensates them into the control loop to eliminate their effects. However, the ESO can only achieve a precise estimation of constant or slowly varying disturbances. When the disturbance is periodically changing, satisfactory results cannot be obtained. In this paper, a generalized high-order extended state observer (GHOESO) is proposed to achieve the precise estimation of known frequency sinusoidal disturbance signals and improve disturbance suppression levels. Through numerical simulations, a traditional ESO and GHOESO are compared in terms of disturbance observation capability and disturbance suppression ability for single and compound disturbances based on our prior knowledge of disturbance frequency. The effectiveness of the proposed GHOESO method is verified. Finally, the algorithm is applied to an airborne optoelectronic stabilization platform for a 1°/1 Hz swing experiment on a space hexapod swing table. The experimental results demonstrate the superiority of the GHOESO proposed in this paper.

## 1. Introduction

**Figure 1:**
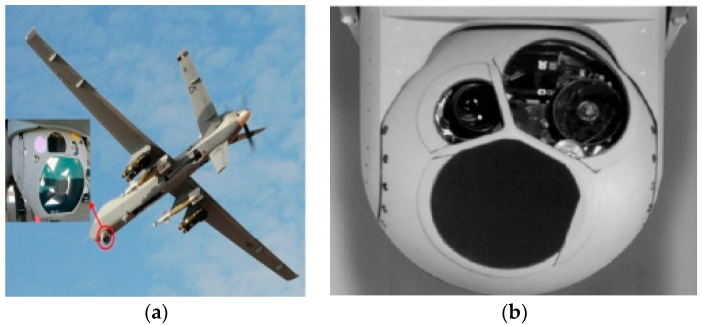
Unmanned aerial vehicles and airborne optoelectronic stability platforms: (**a**) a drone loaded with an optoelectronic device and (**b**) an airborne photoelectric stabilized platform.

The airborne electro-optical stabilization platform is utilized in the airborne environment of unmanned reconnaissance aircraft, transport aircraft and fighter aircraft. It utilizes visible, infrared and laser detectors as sensitive components and employs servo-stabilization control to isolate the attitude changes and disturbances of the aircraft’s spatial motion carrier. This is carried out to maintain the stable orientation of the detector relative to inertial space, thereby achieving rapid capture, tracking, aiming, ranging and striking functions for interested targets through an integrated opto-mechanical-electrical system [1,2,3]. The line-of-sight (LOS) stabilization technology of the electro-optical stabilization platform is essential for realizing the equipment functions mentioned above. LOS refers to line-of-sight pointing, tracking stability and line-of-sight control. When the carrier rotates around its axis, the direction of sight remains unchanged in the inertial coordinate system. The line-of-sight stabilization system must sense deviations from sensors sensitive to line-of-sight and gradually reduce them. As shown in Figure 1, a schematic diagram of an unmanned aerial vehicle carrying an electro-optical stabilization platform is presented, along with detailed images of the platform. Environmental influences, such as friction between axis systems, wind resistance torque from high-speed movement, unbalanced torque during vibration conditions, and coupling between azimuth-pitch frameworks, impose various adverse effects on electro-optical stabilization platforms. Disturbance suppression is a primary issue faced by airborne electro-optical stabilization platforms’ line-of-sight stability. This represents a significant technical challenge that requires urgent resolution. Disturbances are characterized by uncertainty due to a lack of disturbance sensors for direct measurements, which makes it difficult to establish precise mathematical models or provide direct compensation. In addition, a variety of disturbances such as frictional torque, wind resistance torque, winding force torque, and unbalanced force torques between azimuth-pitch frameworks further complicate the situation. These impacts involve non-modeled disturbances, parameter variations, complex nonlinearities, etc., which cannot be effectively addressed using traditional feedback controls.

In practical engineering scenarios, the controlled object is constantly affected by complex disturbances, leading to the continuous variation of its mathematical model. However, precise modeling of the controlled object requires significant human and material resources. In conclusion, the task of establishing an accurate mathematical model is exceedingly challenging. Consequently, it proves difficult for controller design methods that rely on precise mathematical models to achieve optimal performance in practical engineering scenarios. This leads to a disconnect between controller design methods based on precise mathematical models and their actual applications in control engineering. To address these limitations and bridge the gap between the model-based controller design theory and deviation-based controller design methods, an advanced control algorithm known as ADRC has emerged. ADRC was proposed by the Chinese scholar Dr. Jingqing Han in 1995 [4]. This method does not require knowledge of the precise mathematical model of the controlled object. In response to the various sources of disturbances that impact the controlled object, ADRC treats both internal and external disturbances as total disturbances. It utilizes the ESO for estimation and compensation. This approach has been widely applied in servo control, flight control and motor control [5,6,7,8,9,10].

The parameter tuning for nonlinear disturbance rejection is difficult, requiring a significant amount of time for engineering debugging. In order to reduce the difficulty of debugging, Professor Gao Zhiqiang from Cleveland State University in the United States proposed an improved linearized ADRC structure based on the foundation of a nonlinear ADRC structure put forward by Researcher Han Jingqing. The ESO controller’s nonlinear gain combination was changed to a proportional-derivative controller. The improved linear ADRC has advantages such as good disturbance resistance, strong robustness, and high accuracy, and can achieve synchronous estimation of system state and disturbance [11,12,13]. Based on these advantages of LADRC, it is widely used in the field of airborne optoelectronic platforms. The quality of ESO observation is the primary factor affecting ADRC performance and disturbance suppression capability [14]. In ref. [15], a design method for the ESO is provided, with parameter adjustment based on experience. In comparison with the high gain observer, sliding mode observer and ESO, the performance and parameters of the ESO were found to be superior in terms of observation. In recent years, scholars have continuously researched and optimized the extended state observer.

Professor Gao Zhiqiang introduces the concept of bandwidth, which linearizes and parameterizes the nonlinear form of ESO [16] and provides a method for configuring the parameters of LESO, greatly reducing the design parameters of ADRC and making it more convenient for engineering applications. In ref. [17], the convergence of estimation errors of the ESO under the assumption that total disturbances satisfy certain boundedness conditions was analyzed. In refs. [11,18], the estimation performance of both the LESO from the perspectives of known object models and completely unknown models is studied, and they conclude that the observation capability of the LESO is proportional to the observer bandwidth. They also point out that when part of the object model is known, the LESO has better observation effects. In ref. [19], it was concluded that NESO performance gradually decays as frequency increases and improves existing parameter configuration methods.

Professor Jin Kunshan discusses the issue of limited bandwidth of the state observer in conventional ADRC when dealing with multi-source uncertain disturbances that include measurement noise. This limitation results in the issue of reduced accuracy in disturbance estimation and constrained control performance [20]. A strategy for linear ADRC based on cascaded state observers is proposed and validated on a magnetic levitation ball system through experiments. The results demonstrate the effectiveness of cascaded state observers. In ref. [21], all unknown matching/non-matching disturbances were concentrated, and compensated them through extended state observer (ESO) but faced the problem of limited gain when noise is present. Therefore, a model-based disturbance observer was proposed. In refs. [22,23], an adaptive enhanced Kalman filter (AAKF) combined with an extended state observer was proposed to address the issue of controller gain being limited by sensor noise. The output of the AAKF was integrated into backstepping control design to achieve rapid disturbance suppression and noise reduction. Recently, a joint unknown input observer (UIO) was developed to offer the convergent estimations of both the system states and unknown inputs, solving the problems of nonlinear strict-feedback multi-agent systems with both external disturbance and sensor uncertainties [24,25,26]. Scholars have improved the ESO’s gain through filtering and cascading to enhance its anti-disturbance effect. However, the traditional ESO can only accurately estimate aggregated disturbances in a constant form. In airborne settings, aggregated disturbances typically exhibit time-varying and sinusoidal characteristics, making it impractical for the traditional ESO to fully estimate them. To address this issue, in ref. [27], a high-order ESO for estimating time-varying disturbances was used and compared it with the traditional ESO. In ref. [28,29], the author analyzed the performance of a high-order ESO and pointed out that there still existed a sinusoidal error for time-varying sinusoidal disturbances.

As the cornerstone of ADRC, the effectiveness of disturbance observation by the ESO determines the system’s capability to suppress disturbances. However, the traditional ESO is only capable of accurately estimating constant disturbances, and its performance in handling periodic disturbances is subpar. Can the observation effects for periodic disturbances be enhanced by improving the structure of the ESO, rather than simply increasing its gain as commonly pursued in previous studies? In this paper, a generalized high-order extended state observer (GHOESO) is proposed under the assumption of known disturbance frequency in order to achieve precise estimation of periodic disturbances. This paper first analyzes the stability and performance of the traditional ESO through theoretical derivation. Then, it elaborates on the design method of the GHOESO and analyzes its stability. Furthermore, numerical simulations comparing the traditional ESO with the GHOESO are conducted based on mathematical models of airborne electro-optical stabilization platforms. Finally, experimental comparison analysis is carried out using a space hexapod swing table to simulate actual flight conditions, evaluating the stability and accuracy of both control methods.

## 2. Extended State Observer Control

For an n-order single-input single-output (SISO) system, the state equation can be expressed as follows [3]:

(1) $$\left\{\begin{array}{l} {\dot{x}}_{1}=x_{2}\\ {\dot{x}}_{2}=x_{3}\\ \vdots \\ {\dot{x}}_{n-1}=x_{n}\\ {\dot{x}}_{n}=f\left(x_{1},x_{2},\cdots x_{n},w\left(t\right),t\right)+bu\\ y=x_{1} \end{array}\right.$$

where u is the input of the system, y is the output of the system, b is the gain of input, and $f\left(x_{1},x_{2},\cdots x_{n},w\left(t\right),t\right)$ is the lumped disturbance, and it includes the comprehensive influence of internal uncertainty and external interference of the system.

From Equation (1), the extended equation of the state is expressed as follows:

(2) $$\left\{\begin{array}{l} \dot{x}=Ax+Bu+Eh\\ y=Cx \end{array}\right.$$

where $A={\left[\begin{array}{ccccc} 0 & 1 & 0 & \cdots & 0\\ 0 & 0 & 1 & \cdots & 0\\ \vdots & \vdots & \vdots & \ddots & \vdots \\ 0 & 0 & 0 & \cdots & 1\\ 0 & 0 & 0 & 0 & 0 \end{array}\right]}_{\left(n+1\right)\times \left(n+1\right)}$, $B={\left[\begin{array}{c} 0\\ 0\\ \vdots \\ 1\\ 0 \end{array}\right]}_{\left(n+1\right)\times 1}$,$E={\left[\begin{array}{c} 0\\ 0\\ \vdots \\ 0\\ 1 \end{array}\right]}_{\left(n+1\right)\times 1}$,$C={\left[\begin{array}{c} 1\\ 0\\ \vdots \\ 0\\ 0 \end{array}\right]}_{\left(n+1\right)\times 1}$, $h=\frac{df\left(x_{1},x_{2},\cdots x_{n},d\left(t\right),t\right)}{dt}+\frac{dw\left(t\right)}{dt}$.

where $f\left(x_{1},x_{2},\cdots x_{n},d\left(t\right),t\right)$ is the influence of internal uncertainty and $w\left(t\right)$ is the influence of external interference. The fundamental principle of the traditional ESO design theory is to restructure the system by treating the disturbance caused by system uncertainty as an extended state. The lumped disturbance is defined as a new extended state expressed as $x_{n+1}=f\left(x_{1},x_{2},\cdots x_{n},w\left(t\right),t\right)$. The ESO controller can be designed as follows:

(3) $$\left\{\begin{array}{l} {\dot{z}}_{1}=z_{2}-\beta_{1}\left(z_{1}-y\right)\\ {\dot{z}}_{2}=z_{3}-\beta_{2}\left(z_{1}-y\right)\\ \vdots \\ {\dot{z}}_{n}=z_{n+1}-\beta_{n}\left(z_{1}-y\right)+bu\\ {\dot{z}}_{n+1}=-\beta_{n+1}\left(z_{1}-y\right) \end{array}\right.$$

where z is the extended state of observation. According to Equation (3), the ESO controller can be further expressed as follows:

(4) $$\left\{\begin{array}{l} \dot{z}=Az+Bu+L\left(y-\hat{y}\right)\\ \hat{y}=Cz \end{array}\right.$$

where $L={\left[\begin{array}{cccc} \beta_{1} & \beta_{2} & \cdots & \beta_{n+1} \end{array}\right]}^{T}$ is the gain matrix of the ESO controller. The observation error of ESO is defined as e=z−x, and the observation error equation of the ESO is written as

(5) $$\dot{e}=\left(A-LC\right)e-Eh$$

where e is the state observation error. From the definition of matrix A, L, and C, $\left(A-LC\right)$ is the Huiwitz matrix. Under the common influence of internal changes and external disturbance, when the system reaches the equilibrium point, it is approximately 0, which will have a continuous effect in the whole process of the system.

To obtain the state of the above controlled object and the estimated disturbance, the following linear state feedback control rate is adopted to design the ESO controller, as follows [30]:

(6) $$u\left(t\right)=\frac{k_{1}\left(r\left(t\right)-z_{1}\left(t\right)\right)+\cdots k_{n}\left(r^{n-1}\left(t\right)-z_{n}\left(t\right)\right)-z_{n+1}\left(t\right)}{b}$$

where $k_{1},k_{2}\cdots k_{n}$ are the gain of state and r(t) is the value of input signal. Based on the formula above, we can draw several conclusions, as follows:

1. The ESO focuses on estimating system dynamics and disturbances, which can increase the computational burden for online estimation by observers and may result in controller saturation more easily.
2. When the differential of external interference is 0, $\left(A-LC\right)$ is the Hurwitz matrix. The observation error equation of the ESO is $\dot{e}=\left(A-LC\right)e$, where e gradually converges to zero, and it represents the disturbance observation error of the ESO gradually converging to zero. It is concluded that the ESO can effectively eliminate the influence of external constant interference.
3. When the external disturbance derivative is constant, the observation error equation of the ESO has an asymptotic stable equilibrium point: $e_{\infty }={\left(A-LC\right)}^{-1}E\left(dw\left(t\right)/dt\right)$. The estimation of disturbance by the ESO in this case demonstrates a steady-state error, and the magnitude of the observer’s steady-state error increases with the rate of change in external disturbance.
4. For other types of external disturbances, the derivative with respect to time is time-varying, and the observer error converges to a neighborhood whose size depends on the upper bound on the value of $dw\left(t\right)/dt$. For other types of external disturbances, the time derivative is time-varying, and the observer error depends on the upper bound of the value of $dw\left(t\right)/dt$.

In order to simplify the debugging process, Professor Gao Zhiqiang proposed an ESO parameter bandwidth tuning method in ref. [20]. This method takes the second-order system as an example, and the controller gain is adjusted as follows:

(7) $$\left[\begin{array}{ccc} \beta_{1} & \beta_{2} & \beta_{3} \end{array}\right]=\left[\begin{array}{ccc} \omega_{0}\alpha_{1} & \omega_{0}^{2}\alpha_{2} & \omega_{0}^{3}\alpha_{3} \end{array}\right]$$

where $\omega_{0}$ is the bandwidth of the ESO controller, $\alpha_{1},\alpha_{2},\alpha_{3}$ are the parameters of the ESO controller, and $\beta_{i}$ is the characteristic polynomial of $s^{3}+\alpha_{1}s^{2}+\alpha_{2}s+\alpha_{3}$; thus, the original extended equation of state is

(8) $$\left\{\begin{array}{l} {\dot{z}}_{1}=z_{2}-\beta_{1}\left(z_{1}-y\right)\\ {\dot{z}}_{2}=z_{3}-\beta_{2}\left(z_{1}-y\right)\\ {\dot{z}}_{3}=-\beta_{3}\left(z_{1}-y\right) \end{array}\right.$$

The selection of parameters needs to ensure that the following estimation error dynamic Equation (9) satisfies the Hurwitz stability condition.

(9) $$\lambda_{0}\left(s\right)=s^{3}+\alpha_{1}s^{2}+\alpha_{2}s+\alpha_{3}={\left(s+\omega_{0}\right)}^{3}$$

Therefore, there is only one parameter to debug, which greatly simplifies the debugging process. The convergence of the ESO has been demonstrated by Professor Guo’s pattern segment [31]. However, this paper will not prove the convergence of the ESO.

## 3. Proposed Generalized High-Order Extended State Observer

### 3.1. Design of GHOESO

For the system, as shown in Equation (1), $f\left(x_{1},x_{2},\cdots x_{n},w\left(t\right),t\right)$ is the lumped disturbance of the system, including the internal uncertainty of the system and the external interference. The internal uncertainty and the external interference are expressed as $f\left(x_{1},x_{2},\cdots x_{n},t\right)$ and $w\left(t\right)$, respectively. Define the lumped disturbance $d\left(t\right)$ represented as $d(t)=f\left(x_{1},x_{2},\cdots x_{n},w\left(t\right),t\right)$. In the paper, a GHESO is proposed, and the following two assumptions are made:

**Assumption** **1:** *System internal uncertainty function* $f\left(x_{1},x_{2},\cdots x_{n},t\right)$*is global Lipschitz,*$\forall t\geq 0,x={\left[x_{1},x_{2}\cdots x_{n}\right]}^{T},y={\left[y_{1},y_{2}\cdots y_{n}\right]}^{T}$*,*

(10) $$\left|f\left(x_{1},x_{2}\cdots x_{n},t\right)-f\left(y_{1},y_{2}\cdots y_{n},t\right)\right|\leq c\left\|x-y\right\|$$

*where* c *is a Lipschitz constant.*

**Assumption** **2:** *The* r *order derivation of external interference* $d\left(t\right)$ *is bounded, there is a positive constant* δ*, and there is* $\delta =\sup_{t>0}\left|d^{\left(r\right)}(t)\right|$.

Under the premise of Assumption 2, external interference $d\left(t\right)$ can be expanded by Taylor’s formula:

(11) $$d\left(t\right)=\sum_{i=0}^{r-1}a_{i}t^{i}+\xi \left(t\right)$$

where $a_{i}$ is the unknown constant and $\xi \left(t\right)$ is the residual after Taylor expansion.

$d^{\left(i\right)}(t)$ expands to a new state variable, and $x_{n+j}=d^{\left(j-1\right)}\left(t\right),j=1,2\cdots r$; thus, the extended state variable of the GHOESO can be expressed as $z={\left[z_{1},z_{2}\cdots ,z_{n+r}\right]}^{T}$.

The parameter $\beta_{i}\left(i=1,\cdots n+r\right)$ must be selected to satisfy that the polynomial $\lambda^{n+r}+\beta_{1}\lambda^{n+r-1}+\cdots +\beta_{n+r-1}\lambda +\beta_{n+r}$ is a Hurwitz matrix. For sinusoidal periodic disturbance, there is $d\left(t\right)=\sin(\omega t+\varphi )$, where ω is the frequency of the sinusoidal periodic disturbance, and the second-order differential of the sinusoidal periodic disturbance can be indicated as $\ddot{d}\left(t\right)=-\omega^{2}d\left(t\right)$. The GHESO controller is as follows:

(12) $$\left\{\begin{array}{l} \dot{\bar{z}}=\bar{A}\bar{z}+\bar{B}u+\bar{L}\left(y-\hat{y}\right)\\ \hat{y}=\bar{C}\bar{z} \end{array}\right.$$

where $\bar{A}={\left[\begin{array}{cccccc} 0 & 1 & 0 & 0 & 0 & 0\\ 0 & 0 & 1 & 0 & 0 & 0\\ 0 & 0 & 0 & \cdots & 0 & 0\\ 0 & 0 & 0 & \ddots & 1 & 0\\ 0 & 0 & 0 & \cdots & -\omega^{2} & 1\\ 0 & 0 & 0 & \cdots & 0 & 0 \end{array}\right]}_{\left(n+r+1\right)\times \left(n+r+1\right)}$, $\bar{B}={\left[\begin{array}{c} 0\\ 0\\ \vdots \\ 1\\ 0\\ 0\\ 0 \end{array}\right]}_{\left(n+r+1\right)\times 1}$, $\bar{L}={\left[\begin{array}{ccccccc} \beta_{1} & \beta_{2} & \cdots & \beta_{n} & \beta_{n+1} & \cdots & \beta_{n+r+1} \end{array}\right]}_{\left(n+r+1\right)\times 1}^{T}$, $\bar{C}={\left[\begin{array}{c} \begin{array}{c} \begin{array}{ccccccc} 1 & 0 & \cdots & 0 & 0 & 0 & 0 \end{array} \end{array} \end{array}\right]}_{1\times \left(n+r+1\right)}$.

### 3.2. GHOESO Stability Analysis

Based on the stability analysis of the GHESO proposed above, the system state error equation is obtained based on the extended state designed by Equation (11), as follows:

(13) $$\dot{e}=\left(A-LC\right)e-Eh=A_{0}e-d_{0}$$

where $A_{0}=\left(\bar{A}-\bar{L}\bar{C}\right)$ is the Hurwitz matrix and $d_{0}=d\left(d\left(t\right)\right)/dt$. There is a positive definite matrix ${A_{0}}^{T}P+PA_{0}=-I_{\left(n+r\right)\left(n+r\right)}$, and considering the Lyapunov function of the quadratic form $V_{1}\left(e\right)=e^{T}Pe$, $V_{1}\left(e\right)$ satisfies the following inequality:

(14) $$\lambda_{\min}\left(P\right){\left\|e\right\|}^{2}\leq V_{1}\left(e\right)\leq \lambda_{\max}\left(P\right){\left\|e\right\|}^{2}$$

(15) $$\left\|\frac{\partial V_{1}\left(e\right)}{\partial e}\right\|=\left\|2e^{T}P\right\|\leq 2\left\|P\right\|\left\|e\right\|=2\lambda_{\max}\left(P\right)\left\|e\right\|$$

$\lambda_{\min}\left(P\right){\left\|e\right\|}^{2}$ and $\lambda_{\max}\left(P\right){\left\|e\right\|}^{2}$ represent the smallest and largest eigenvalues of the symmetric matrix, and

(16) $$V_{1}\left(e\right)=-Le^{T}e+2{d_{0}}^{T}Pe$$

According to Assumptions 1 and 2:

(17) $$\left\|d_{0}\right\|=\left|\frac{1}{\beta^{n+r-1}}e^{\left(r\right)}\left(t\right)\right|\leq \frac{\delta }{\beta^{n+r-1}}$$

Substituting (17) into (16), we can obtain the following:

(18) $${\dot{V}}_{1}\left(e\right)\leq -\left(1-\theta \right)\left(1-\frac{2\lambda_{\max}\left(P\right)c}{\beta^{n-1}}\right){\left\|e\right\|}^{2}-M(e)$$

where 0<θ<1, $M(e)=\left[1-\frac{2\lambda_{\max}\left(P\right)c}{\beta^{n-1}}\theta \left\|e\right\|-\frac{2\lambda_{\max}\left(P\right)\delta }{\beta^{n+r-1}}\right]\left\|e\right\|$; thus, we can obtain the following:

(19) $$\forall \left\|e\right\|\geq \frac{2\lambda_{\max}\left(P\right)\delta }{\theta \beta^{r}\left[\beta^{n}-2\lambda_{\max}\left(P\right)c\right]}=\mu >0$$

where $M(e)=N\left(e\right)\left\|e\right\|\geq 0$, and Equation (16) satisfies the following conditions:

(20) $${\dot{V}}_{1}\left(e\right)\leq \left(1-\theta \right)\left(1-\frac{2\lambda_{\max}\left(P\right)c}{\beta^{n-1}}\right){\left\|e\right\|}^{2}$$

Eventually, the observation error converges to an interval less than $\mu \sqrt{\frac{\lambda_{\max}\left(P\right)}{\lambda_{\min}\left(P\right)}}$.

## 4. Simulation Based on Airborne Photoelectric Platform Model

### 4.1. Model of Airborne Photoelectric Stabilization Platform

The dynamic models of the two-axis two-frame structure of the airborne photoelectric stabilization platform and the motor voltage equation are shown in Equations (21) and (22), respectively, as shown in [3].

(21) $$\left\{\begin{array}{l} T_{x}\left(t\right)=K_{x}I_{x}\left(t\right)=J_{x}{\ddot{\theta }}_{x}\left(t\right)\\ T_{y}\left(t\right)=K_{y}I_{y}\left(t\right)=J_{y}{\ddot{\theta }}_{y}\left(t\right) \end{array}\right.$$

(22) $$\left\{\begin{array}{l} u_{x}=R_{x}I_{x}+L_{x}\frac{dI_{x}}{dt}+C_{ex}{\dot{\theta }}_{x}\\ u_{y}=R_{y}I_{y}+L_{y}\frac{dI_{y}}{dt}+C_{ey}{\dot{\theta }}_{y} \end{array}\right.$$

where $T_{x}\left(t\right)$, $K_{x}$, $I_{x}\left(t\right)$, $J_{x}$, and ${\ddot{\theta }}_{x}\left(t\right)$ are the motor torque, torque coefficient, rotational inertia, and angular speed of the azimuth motor, respectively. $T_{y}\left(t\right)$, $K_{y}$, $I_{y}\left(t\right)$, $J_{y}$, and ${\ddot{\theta }}_{y}\left(t\right)$ are the motor torque, torque coefficient, rotational inertia, and angular speed of the pitch motor, respectively. Since the mathematical models for the azimuth axis and pitch axis are identical, as well as the control methods, with only differences in parameters, this paper focuses solely on studying the mathematical model for the azimuth axis. However, it should be noted that the mathematical model is also applicable to the pitch axis. In the previous analysis, ignoring the influence of self-inductance and back potential, the transfer function of the control system is a first-order pure integral, expressed as

(23) $$G\left(s\right)=\frac{K_{x}}{J_{x}R_{x}}\cdot \frac{1}{s}$$

where $k=\frac{K_{x}}{J_{x}R_{x}}$; thus, the above formula becomes the following:

(24) $$G\left(s\right)=\frac{k}{s}$$

**Figure 2:**
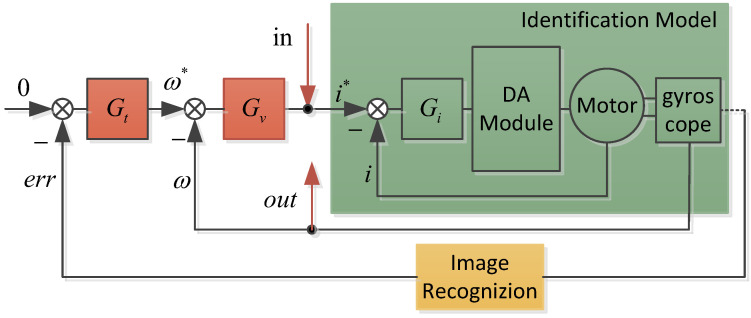
Sweep frequency modeling control logic diagram.

**Figure 3:**
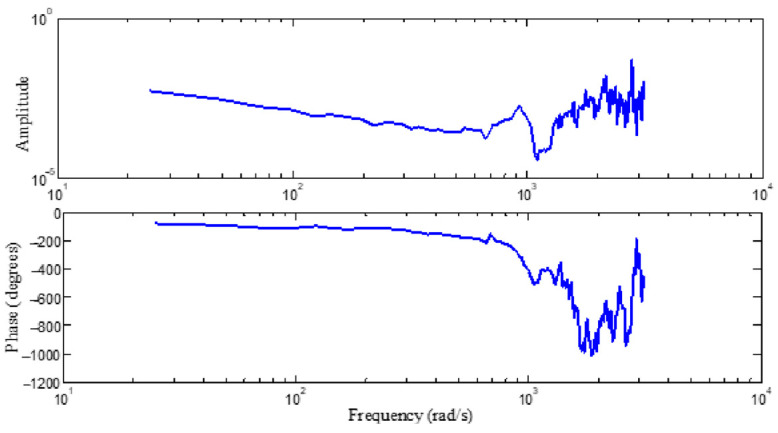
Transfer function of the control object for sweep frequency identification.

The parameters in the above formula are determined through a frequency sweep experiment. The relationship between the injection and output of the frequency sweep signal is shown in Figure 2: “in” is the injection point of the sweep signal, “out” is the output collection point. The sweep injection consists of a sine wave with varying frequencies. The characteristic curve of the identified model is determined as shown in Figure 3, which can be determined from the following model: k=0.0023. Subsequent simulations and experiments are conducted on the basis of this model.

### 4.2. Application of Proposed GHOESO

The system equation is

(25) $$\dot{y}=k\cdot u+d$$

where u is the input of the system, d is lumped disturbance, y is the output, and $y=\dot{x}$:

(26) $$\left\{\begin{array}{l} \dot{x}=k\cdot u+d\\ y=x \end{array}\right.$$

The system equation is as follows:

(27) $$\left\{\begin{array}{l} {\dot{x}}_{1}=x_{2}+k\cdot u\\ {\dot{x}}_{2}=d\\ y=x_{1} \end{array}\right.$$

The linear ESO bandwidth theory is used to design the ESO controller, as follows:

(28) $$\left\{\begin{array}{l} {\dot{z}}_{1}=z_{2}+k\cdot u-\beta_{1}\left(z_{1}-y\right)\\ {\dot{z}}_{2}=-\beta_{2}\left(z_{1}-y\right)\\ u=\frac{1}{k}\left[-{\dot{z}}_{2}+\alpha_{1}\left(y-z_{1}\right)\right] \end{array}\right.$$

where z is the state observation value, $\beta_{1}$ and $\beta_{2}$ indicate the gain of the ESO, and $\alpha_{1}$ indicates the gain of control.

The GHOESO algorithm reconstructs the system based on the frequency of the external sinusoidal disturbance. For sinusoidal signals with known prior frequency information, the following formula is applicable:

(29) $$\ddot{d}\left(t\right)=-\omega^{2}\cdot d\left(t\right)$$

where ω is the frequency of the disturbance. Define the system expansion state as $x_{2}≜d\left(t\right)$, $x_{3}≜\dot{d}\left(t\right)$, and $x_{4}≜\ddot{d}\left(t\right)+\omega^{2}\cdot x_{2}$; thus, the equation of state can be written as follows:

(30) $$\left\{\begin{array}{l} {\dot{x}}_{1}=x_{2}+k\cdot u\\ {\dot{x}}_{2}=x_{3}\\ {\dot{x}}_{3}=x_{4}-\omega^{2}\cdot x_{2}\\ {\dot{x}}_{4}=h\left(t\right)\\ y=x_{1} \end{array}\right.$$

The GHOESO controller is designed as follows:

(31) $$\left\{\begin{array}{l} {\dot{z}}_{1}=z_{2}+k\cdot u-\beta_{1}\left(z_{1}-y\right)\\ {\dot{z}}_{2}=z_{3}-\beta_{2}\left(z_{1}-y\right)\\ {\dot{z}}_{3}=z_{4}-\omega^{2}\cdot z_{2}-\beta_{3}\left(z_{1}-y\right)\\ {\dot{z}}_{4}=-\beta_{4}\left(z_{1}-y\right) \end{array}\right.$$

### 4.3. Simulation Results

#### 4.3.1. One-Hertz Single Sinusoidal Disturbance

**Figure 4:**
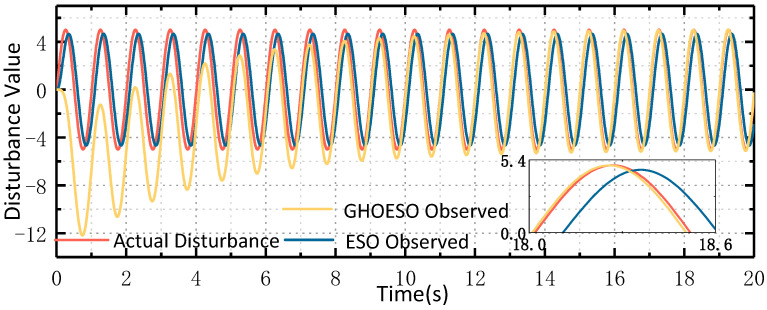
Comparison of ESO and GHOESO observational disturbances under 1 Hz disturbance.

**Figure 5:**
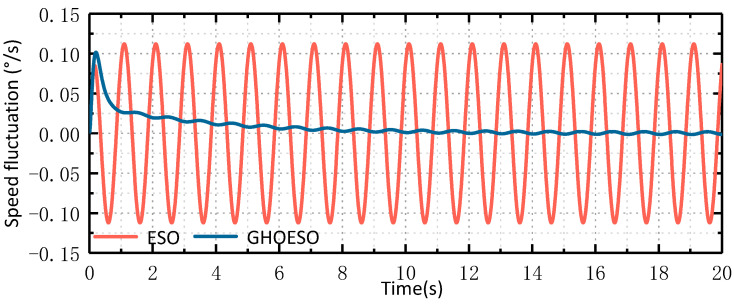
Comparison of ESO and GHOESO velocity fluctuations under 1 Hz disturbance.

The simulation sets the disturbance as a sinusoidal disturbance with a frequency of 1 Hz and an amplitude of 5. In the practical application of airborne platforms, disturbances impact the platform in the form of torque. The unit of torque is Nm, but there is no suitable sensor available to directly measure these disturbances. Therefore, in order to simulate the effect of actual disturbance on the control system, when the value of d is 5, the simulated current aligns with the actual change in the current. The disturbance d has no unit and solely represents its impact on the control system. The input of the speed loop is 0°/s, and the simulation parameters are set as shown in Table 1. When the sine disturbance is 1 Hz, the observed disturbances and velocity fluctuations of the ESO and the GHOESO are compared, and the simulation waveforms are shown in Figure 4 and Figure 5, respectively. 

As depicted in Figure 4 and Figure 5, When the traditional ESO observes a sinusoidal disturbance with a frequency of 1 Hz, there is a steady-state error. The amplitude of the observed disturbance is 4.7, and there is a certain hysteresis phenomenon. Additionally, the velocity fluctuation is about ±0.11°/s. The observed perturbations of GHOESO are in good agreement with the actual perturbations, exhibiting an amplitude of 5. Furthermore, there is no presence of steady-state error or hysteresis commonly associated with traditional ESO. The velocity fluctuation of GHOESO is approximately ±0.002°/s. When faced with low-frequency sinusoidal perturbations at 1 Hz, GHOESO outperforms traditional ESO observations. The following data is summarized and compared for further analysis.

It can be observed from Table 2. that, given a known disturbance frequency, the performance of the GHOESO is significantly enhanced in comparison to the ESO. When the amplitude of perturbation is 5, the ESO observation error is 0.3, and the GHOESO observation error is reduced to less than 0.01. When the speed loop input is 0°/s, the ESO speed fluctuation is 0.11°/s, and the GHOESO reduces the speed fluctuation to 0.002°/s. The effectiveness of the GHOESO to deal with a single low-frequency disturbance is verified.

#### 4.3.2. Three-Hertz Single Sinusoidal Disturbance

**Figure 6:**
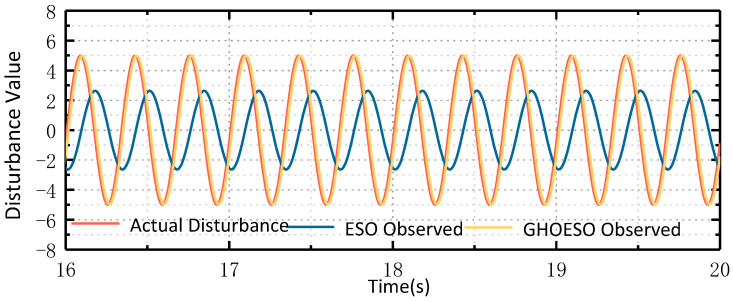
Comparison of ESO and GHOESO observation disturbances under 3 Hz disturbance.

**Figure 7:**
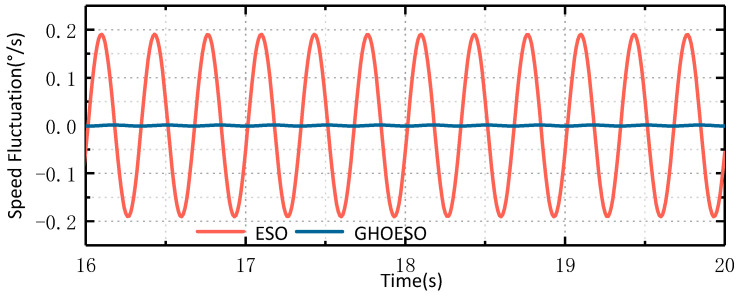
Comparison of ESO and GHOESO speed fluctuations under 3 Hz disturbance.

In order to compare the disturbance observation ability and disturbance suppression ability of the ESO and the GHOESO at higher frequencies, the velocity loop input is kept at 0, and the disturbance is set to sinusoidal disturbance with an amplitude of 5 and a frequency of 3 Hz. At this frequency, simulation parameters are set as shown in Table 3. When the sine disturbance is at 3 Hz, the observed disturbance and velocity fluctuation of the ESO and the GHOESO are compared. The simulation waveforms are presented in Figure 6 and Figure 7, respectively.

As depicted in Figure 6 and Figure 7, when the frequency of disturbance is increased to 3 Hz, the amplitude of sine disturbance observed by the ESO is approximately 2.8, exhibiting a significant hysteresis phenomenon. With an increase in the disturbance frequency, the steady-state error of ESO observation also increases. The hysteresis phenomenon becomes more pronounced, leading to a deterioration in the observation effect. Under the influence of a 3 Hz disturbance, the steady-state error and hysteresis of the GHOESO observation effect are minimal. Furthermore, the ability of GHOESO observation disturbance does not decrease with an increase in frequency. The sinusoidal perturbations of the GHOESO at different frequencies are observed more accurately, and this advantage becomes more obvious as the frequency of perturbations increases. Table 4 below compares the disturbance inhibition ability of the ESO and the GHOESO.

For a disturbance frequency of 3 Hz, it is evident from the table that the inhibition ability of the ESO decreases as the disturbance frequency increases. When the controller parameter and disturbance amplitude remain constant, increasing the frequency from 1 Hz to 3 Hz results in an observed disturbance error of the ESO increasing to 2.2 and velocity fluctuation increasing to 0.2°/s. However, under the known frequency, the increase in frequency does not affect the identification effect of the GHOESO, thus once again confirming the superiority of the GHOESO.

#### 4.3.3. Comparison of Noise Immunity Performance under Compound Disturbance

Due to the complex sources of disturbance on the airborne photoelectric platform, there are various types of disturbances in addition to sinusoidal disturbance torque. These include low-frequency winding torque, motor groove torque, and high-frequency sensor fluctuations with small values. In this section, we simulate multiple forms of complex sinusoidal disturbances and compare the ability of the ESO and the GHOESO to observe these disturbances under complex conditions. The velocity loop input is set at 0, and the disturbance is compounded by superimposing sinusoidal disturbances. The form of disturbance is set as follows:

(32) $$d=d_{1}+d_{2}+d_{3}=A_{1}\sin\left(\omega_{1}t\right)+A_{2}\sin\left(\omega_{2}t\right)+A_{3}\sin\left(\omega_{3}t\right)$$

where $\omega_{i}=2\pi f_{i}$. Set the compound disturbance and parameter settings as illustrated in Table 5 and Table 6 below, respectively.

**Figure 8:**
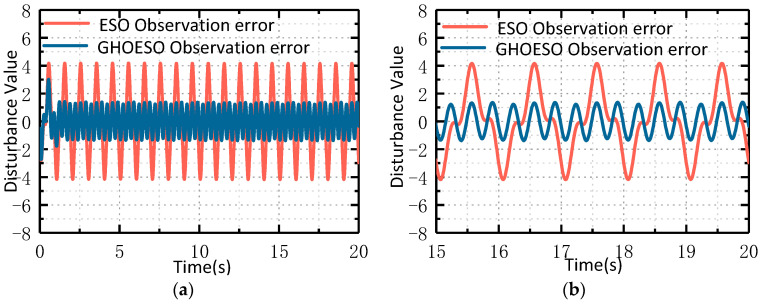
Comparison of ESO and GHOESO observation disturbance and actual disturbance under compound disturbance: (**a**) overall graph; (**b**) local graph.

**Figure 9:**
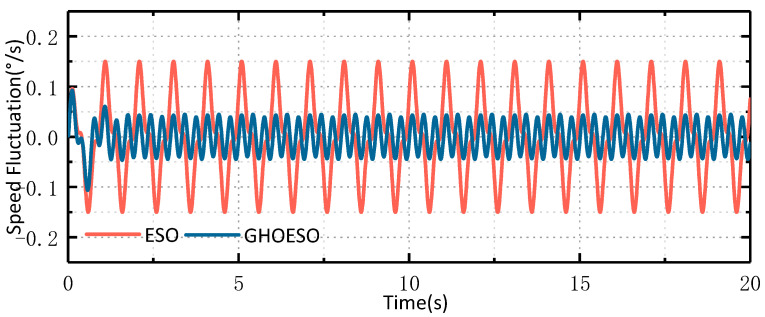
Comparison of ESO and GHOESO speed fluctuations under compound disturbance.

It can be seen from Figure 8 and Figure 9 that the GHOESO for complex sinusoidal superposition disturbance can observe disturbance and smaller velocity fluctuation values more accurately, as shown in Table 7 below.

Under compound disturbance, the performance of both the ESO and the GHOESO is decreased. The disturbance residual of the ESO is 4, the velocity fluctuation is 0.15°/s, and the performance of the GHOESO is decreased. However, for complex disturbance, the performance of the GHOESO is still better than that of the traditional ESO, with a disturbance error of 1.6 and a velocity fluctuation of 0.045°/s.

From the above simulation results, we can draw the following conclusions:

1. Under a single sinusoidal disturbance frequency, ESO observation will exhibit a steady-state error and phase lag. As the disturbance frequency increases, the steady-state error and phase lag become more pronounced, leading to inadequate suppression of disturbances by the ESO. Furthermore, the effectiveness of disturbance suppression weakens as the disturbance frequency increases. When the system is exposed to periodic time-varying external disturbances, it will be influenced by these disturbances. The traditional ESO is unable to completely eliminate the impact of the disturbance. Additionally, the estimation error of the ESO increases as the disturbance period increases, and it decreases as the observer bandwidth increases. However, GHOESO processing exhibits a very small steady-state error and phase lag. Furthermore, the effectiveness of disturbance observation and suppression does not diminish with an increase in the disturbance frequency.
2. The impact of ESO observation disturbance and ESO suppression disturbance is significantly reduced when subjected to the complex disturbance of multiple sinusoidal superposition. The performance of the GHOESO in handling such disturbances will also be compromised, particularly at the peak of the disturbance wave, where the maximum observation error may occur. Nevertheless, the GHOESO still outperforms ESO in managing multiple sinusoidal superpositions.

## 5. Experiment

**Figure 10:**
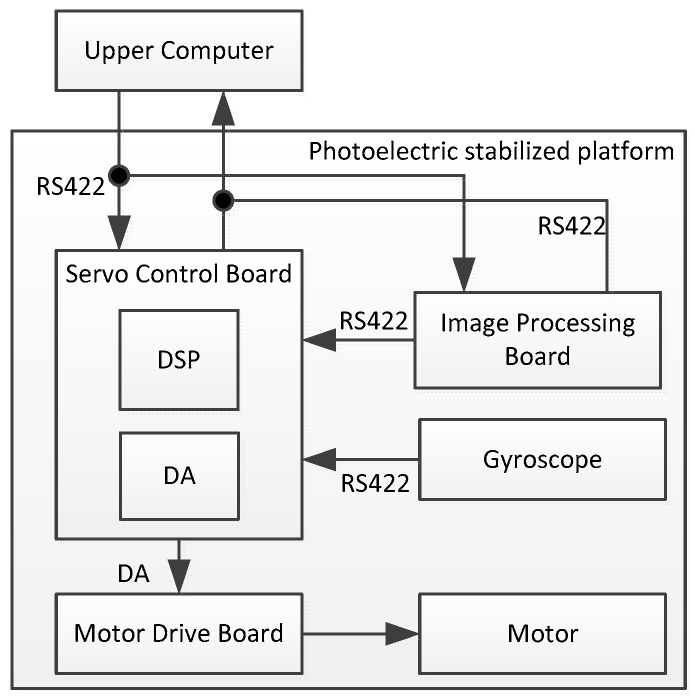
Experimental hardware connection logic block diagram.

In addition to the servo system, the airborne optoelectronic stabilization platform should also include the host computer, image processing board, and other systems. The overall logic block diagram of the platform is shown in Figure 10.

**Figure 11:**
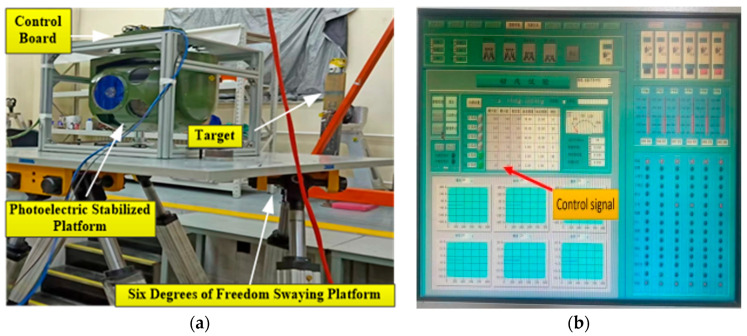
Laboratory experimental equipment and its driving signal settings: (**a**) the six degrees of freedom swaying platform and (**b**) the upper computer and control signals.

The upper computers are connected to the servo system through the RS422 serial port. It sends working instructions to the servo system, which in turn returns to the working state of the upward computer. The image processing board is capable of real-time recognition of the target. When the platform tracks the target, the image processing board sends the current miss distance to the servo system. This distance represents the number of pixels from the center of the target offset field of view. According to the miss distance, the servo system calculates the speed at which the gyro speed ring should pull the target into the center of the field of view. During the tracking process of an airborne optoelectronic stabilized platform, external interference and environmental changes may cause the target to deviate from the center of the field of view. A smaller miss distance, indicating a shorter distance from the center of visual axis to target, results in a higher stability accuracy of the visual axis and a better disturbance suppression ability of the algorithm. The experimental environment is depicted in Figure 11 below.

**Figure 12:**
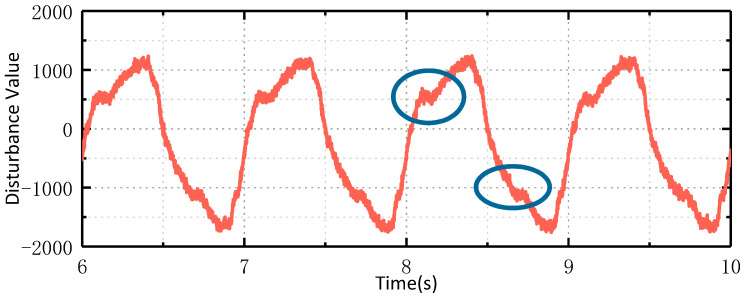
Disturbance observed by ESO when the swaying platform moved at 1°/1 Hz.

**Figure 13:**
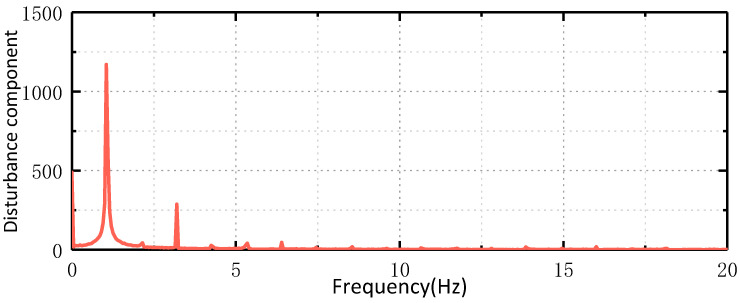
FFT of disturbance observed by ESO.

The experimental equipment and platform consist of a photoelectric platform fixed on the hexapod swing table plane for testing. The rocking table is driven by six motors to control the movement of six degrees of freedom in space, including translation and rotation in orientation, pitch, and roll. This allows for simulating the motion of an aircraft on the platform. The first experiment is the speed ring stabilization experiment. The airborne photoelectric stabilization platform is fixed on the hexapod swinging platform. The platform maintains the stability of the speed ring, while the hexapod swinging platform swings at 1°/1 Hz with two degrees of freedom. The traditional ESO is utilized to observe the disturbance applied to the photoelectric stable platform, and the obtained disturbance curve is shown in Figure 12. The observed disturbance is decomposed into multiple sinusoidal superpositions via Fourier decomposition, as illustrated in Figure 13.

As shown in Figure 12, when the space hexapod swing table moves at 1°/1 Hz, the motion is applied to the platform through the coupling relationship of the frame, and the frequency of the disturbance is also 1 Hz. The circles in the figure represent the Stribeck characteristics of friction force. Fourier decomposition of the disturbance signal shows that the disturbance mainly includes a constant component, 1 Hz sinusoidal component, and 3 Hz sinusoidal component. The following experiments are conducted to verify whether the GHOESO is superior to the traditional ESO in handling sinusoidal disturbance.

**Figure 14:**
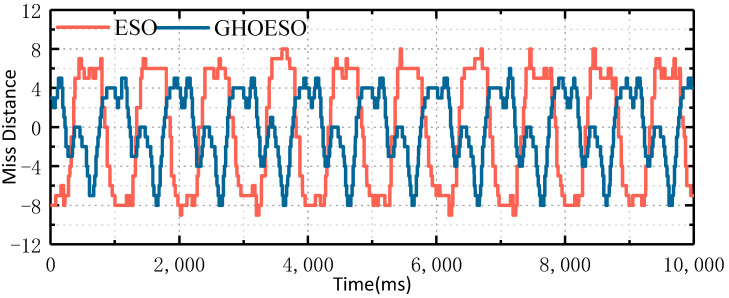
Comparison of miss distance in azimuth direction of ESO and GHOESO.

**Figure 15:**
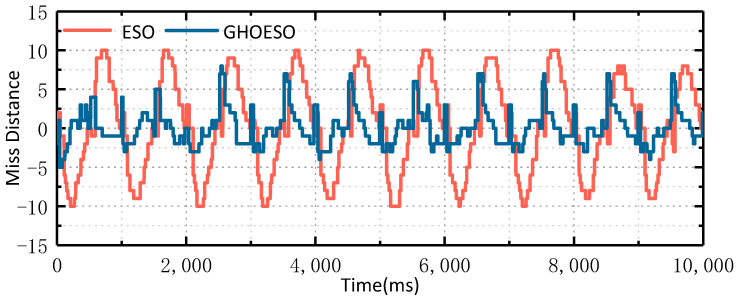
Comparison of miss distance in pitch direction of ESO and GHOESO.

Then, the video tracking experiment was carried out. The field of view of the experimental platform was selected as a small field of view, a parallel optical tube was placed on the target to simulate the target at infinity, the photoelectric platform was controlled to enter the image tracking mode, the optical tube of the target was tracked, and the rocking platform was controlled to move according to the set swaying condition of 1°/1 Hz to simulate the movement of the carrier. The software calculates the set motion trajectory into the control quantity of six motors and controls the cyclic reciprocating motion of the platform through the hydraulic mechanism. Through one serial port of the servo control board, the miss distance data during the experiment are output. The misses in the platform azimuth and pitch direction are shown in Figure 14 and Figure 15.

**Figure 16:**
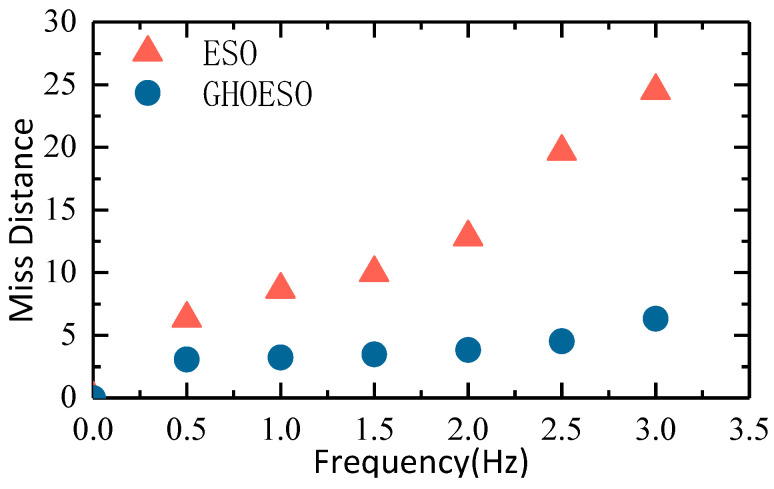
Comparison chart of ESO and GHOESO total miss distance.

As shown in Table 8, at 1°/1 Hz, the ESO control algorithm is used to achieve the fluctuation amplitude of a miss range of ±9 in the azimuth direction and ±10 in the pitch direction. By using the GHOESO control algorithm, the miss amplitude is ±6 in the azimuth direction and ±8 in the pitch direction. Miss distance using the GHOESO control algorithm is reduced in the azimuth and pitch directions. The miss distance standard value decreases from 6.02 to 2.71 in the azimuth direction and from 6.23 to 2.94 in the pitch direction. Thus, the total miss distance can be defined as

(33) $$\gamma =\sqrt{\alpha^{2}+\beta^{2}}$$

where α and β are the standard values of miss distance in the azimuth and pitch directions, respectively. At 1°/1 Hz, the GHOESO reduces the total miss distance from 8.66 to 3.99, which is a reduction of 53.93%. The total miss distance at an amplitude of 1° and a frequency of 0.5 Hz to 3 Hz was further tested, as shown in Figure 16.

Figure 16 illustrates that the traditional ESO exhibits effective anti-disturbance performance at low frequencies. However, as the disturbance frequency increases, its observation effect gradually diminishes, confirming the inability to accurately estimate periodic sinusoidal interference. Specifically, when the disturbance frequency is low, the ESO demonstrates strong effectiveness; however, as the disturbance frequency rises, its ability to suppress disturbances decreases. In contrast, the GHOESO outperforms the ESO in suppressing disturbances when the disturbance frequency is known and maintains this capability regardless of increasing disturbance frequencies.

## 6. Conclusions

This paper analyzes the precise estimation capability of the ESO for constant and slowly varying disturbances through a theoretical derivation. It is observed that when the disturbance takes the form of a sinusoidal periodic variation, the ESO exhibits poor disturbance suppression ability. Even increasing the gain of the ESO does not lead to satisfactory results. To address this issue, a GHOESO control method is proposed in this paper, which can achieve an accurate estimation of known frequency sinusoidal periodic signals. A comprehensive comparison is made between the GHOESO and the traditional ESO in terms of disturbance observation capability and disturbance suppression ability under single 1 Hz and 3 Hz sinusoidal disturbances as well as composite superimposed disturbances. Finally, experiments are conducted on a space hexapod swing table based on an airborne electro-optical stabilization platform. The results demonstrate that the proposed GHOESO improves the observation effect by 53.93% at 1°/1 Hz, and its observation effect does not vary with an increase in sinusoidal frequency.

## Figures and Tables

**Table 1:** Simulation parameter settings under 1 Hz single disturbance.

Parameter	ESO	GHOESO
$\alpha_{1}$	10,000	10,000
$\beta_{1}$	30	30
$\beta_{2}$	300	300
$\beta_{3}$	/	30
$\beta_{4}$	/	300
$\omega^{2}$	/	40

**Table 2:** Comparison of ESO and GHOESO performance under 1 Hz disturbance.

	Disturbance Error	Velocity Fluctuation
ESO	0.3	0.11
GHOESO	<0.01	0.002

**Table 3:** Parameter settings under a single disturbance of 3 Hz.

Parameter	ESO	GHOESO
$\alpha_{1}$	10,000	10,000
$\beta_{1}$	30	30
$\beta_{2}$	300	300
$\beta_{3}$	/	30
$\beta_{4}$	/	300
$\omega^{2}$	/	300

**Table 4:** Comparison of ESO and GHOESO performance under 3 Hz disturbance.

	Disturbance Error	Velocity Fluctuation
ESO	2.2	0.2
GHOESO	<0.01	0.002

**Table 5:** Compound disturbance is set as follows.

	Amplitude	Frequency
Disturbance 1	5	1 Hz
Disturbance 2	1	3 Hz
Disturbance 3	0.01	100 Hz

**Table 6:** Controller parameter setting under compound disturbance.

Parameter	ESO	GHOESO
$\alpha_{1}$	10,000	10,000
$\beta_{1}$	30	30
$\beta_{2}$	300	300
$\beta_{3}$	/	30
$\beta_{4}$	/	300
$\omega^{2}$	/	43.5

**Table 7:** Comparison of ESO and GHOESO under compound disturbance.

	Disturbance Error	Velocity Fluctuation
ESO	4	0.15°/s
GHOESO	1.6	0.045°/s

**Table 8:** One-hertz miss distance in azimuth and pitch direction.

	Azimuth		Pitch	
	Fluctuation Range	Std	Fluctuation Range	Std
ESO	±9	6.02	±10	6.23
GHOESO	±6	2.71	±8	2.94

## Data Availability

Data are contained within this article.
